# Protective Effect of Gadolinium Chloride on Early Warm Ischemia/Reperfusion Injury in Rat Bile Duct during Liver Transplantation

**DOI:** 10.1371/journal.pone.0052743

**Published:** 2013-01-14

**Authors:** Biao Wang, Qi Zhang, Bili Zhu, Zhonglin Cui, Jie Zhou

**Affiliations:** 1 Department of Hepatobiliary Surgery, Nanfang Hospital, Southern Medical University, Guangzhou, People's Republic of China; 2 Liver Transplantation Center, The Third Affiliated Hospital of Sun Yat-sen University, Guangzhou, People's Republic of China; 3 Huiqiao Department, Nanfang Hospital, Southern Medical University, Guangzhou, People's Republic of China; University of Southern California, United States of America

## Abstract

**Background:**

Activation of Kupffer cell (KC) is acknowledged as a key event in the initiation and perpetuation of bile duct warm ischemia/reperfusion injury. The inhibitory effect of gadolinium chloride (GdCl_3_) on KC activation shows potential as a protective intervention in liver injury, but there is less research with regard to bile duct injury.

**Methods:**

Sixty-five male Sprague-Dawley rats (200–250 g) were randomly divided into three experimental groups: a sham group (*n* = 15), a control group (*n* = 25), and a GdCl_3_ group (*n* = 25). Specimen was collected at 0.5, 2, 6, 12 and 24 h after operation. Alanine aminotransferase (ALT), alkaline phosphatase (ALP) and total bilirubin (TBIL) of serum were measured. Tumor necrosis factor-α (TNF-α), Capase-3 activity and soluble Fas (sFas) were detected. The pathologic changes of bile duct were observed. Immunochemistry for bile duct Fas was performed. Apoptosis of bile duct cells was evaluated by the terminal UDP nick end labeling assay.

**Results:**

GdCl_3_ significantly decreased the levels of ALT, ALP and TBIL at 2, 6, 12, and 24 h, and increased serum sFas at 2, 6 and 12 h (*P*<0.05). TNF-α was lower in the GdCl_3_ group than in the control group at 2, 6, 12 and 24 h (*P*<0.05). Preadministration of GdCl_3_ significantly reduced the Caspase-3 activity and bile duct cell apoptosis at 2, 6, 12 and 24 h. After operation for 2, 6 and 12 h, the expression of Fas protein was lower in the GdCl_3_ group than in the control group (*P*<0.05).

**Conclusions:**

GdCl_3_ plays an important role in suppressing bile duct cell apoptosis, including decreasing ALT, ALP, TBIL and TNF-α; suppressing Fas-FasL-Caspase signal transduction during transplantation.

## Introduction

Biliary complications remain a major cause of morbidity and mortality after orthotopic liver transplantation [1], [2]. Hepatic artery thrombosis [3], prolonged cold injury time [1], [2], [4], warm ischemia/reperfusion injury [1], [4], [5], and immunological rejection [6], [7] have all been variously associated with structural changes and functional lesions of the biliary tract following grafting. Among these pathogenic factors, warm ischemia/reperfusion injury is recognized as the major cause of the early phase of biliary lesion development [1], [8].

The process of biliary tract warm ischemia/reperfusion injury is a cascade of inflammatory events involving multiple interconnected factors [1], [2], [4], [9]. Recent studies have indicated that activated Kupffer cell (KC) release a large amount of proinflammatory cytokines, such as reactive oxygen species, tumor necrosis factor-α (TNF-α), and proteases during the initial phase of warm ischemia/reperfusion injury [10], [11], [12]. The interaction of cytokines causing neutrophil adherence, disturbance of the biliary tract microcirculation, and bile duct cell apoptosis lead to the nonfunctioning epithelium [9], [11], [13]. As a consequence, activation of KC has been identified as a critical event in the initiation and perpetuation of bile duct warm ischemia/reperfusion injury. To attenuate inflammation, substances that inhibit KC activity have been characterized. Gadolinium chloride (GdCl_3_), a rare earth metal salt with marked similarity to calcium salts in regard to crystal radii, can potentially inhibit the phagocytic and proteolytic activation of KC by interfering with calcium uptake and calcium-dependent cellular processes [10], [14]. Although GdCl_3_ has proved effective in preventing alcoholic liver injury [15], minimizing ischemia/reperfusion liver injury [10], [16], and attenuating endotoxemia [17], direct effects on bile duct ischemia/reperfusion injury after liver transplantation are rare. Our laboratory has established an orthotopic autologous rat liver transplantation model [18]. This model simulates the entire process of clinical liver transplantation and avoids the effects of infection and immune suppression while accurately controlling bile duct warm ischemia/reperfusion time. This model is therefore suitable for investigation of the effect of GdCl_3_ on rat bile duct early warm ischemia/reperfusion injury during transplantation.

## Materials and Methods

### Materials

Sixty-five male Sprague-Dawley rats (200–250 g) were obtained from Southern Medical University Animal Center, Guangzhou, China. All animals received humane care in compliance with the European Convention on Animal Care. All surgical procedures were approved by the Southern Medical University Animal Care and Use Committee. GdCl_3_ was purchased from Sigma-Aldrich Co., LLC (USA). Serum TNF-α and soluble Fas (sFas) were measured by ELISA kits supplied by R&D systems, Inc. (USA). Caspase-3 activity was measured using Caspase-3 Activity Assay kit (Beyotime Institute of Biotechnology, Nantong, Jiansu, China). The terminal UDP nick end labeling (TUNEL) assay kit was supplied by Roche Diagnostics Corporation (USA).

### Experimental Groups

The rats were randomly divided into three experimental groups: (1) a sham group (*n* = 15), which underwent laparotomy and liver dissection without liver transplantation; (2) a GdCl_3_ group (*n* = 25), which was pretreated with GdCl_3_ before orthotopic autologous liver transplantation; and (3) a control group (*n* = 25), which was administered an equivalent volume of 0.9% normal saline before orthotopic autologous liver transplantation. GdCl_3_ was injected intravenously into the tail at a dose of 10 mg/kg body weight 48 and 24 h before surgery. The rats were kept under a 12 h/12 h light/dark cycle, had free access to rat chow and water (Animal Center, Southern Medical University, Guangzhou, China), and were fasted for 8 h before surgery. The rats were sacrificed at 0.5, 2, 6, 12 and 24 h after surgery for the measurement of serum transaminase (alanine aminotransferase [ALT], total bilirubin [TBIL] and alkaline phosphatase [ALP]), TNF-α, Caspase-3 activity and sFas levels, performance of the TUNEL assay and Pathological examination, and determination of the Fas protein expression.

### Surgical Procedures

The orthotopic autologous liver transplantation model was induced according to methods previously described [18]. Under ether anesthesia, the abdomen was opened through an inverted T-incision, and the left diaphragmatic, hepatoesophageal ligament, and right adrenal veins were separated and ligated. The suprahepatic and infrahepatic inferior vena cava was anatomized, and the common bile duct, portal vein, and celiac trunk were separated over the margin of the duodenal bulb. After clipping the distal portal vein and celiac trunk with bulldog clamps, lactated Ringer's solution (4°C, including 20 ml heparin 12.5 U/ml) was simultaneously injected into both the portal vein and coeliac trunk at the rate of 2.5 ml/min to perform cold perfusion with transfixion pins. Subsequently, the clamps were placed on the suprahepatic and infrahepatic inferior vena cava, and an incision was placed above the clipped area in the infrahepatic inferior vena cava as an outflow tract. The cold perfusion phase was terminated when the normal liver color faded. The portal vein, celiac trunk, and infrahepatic inferior vena cava outflow tract were then repaired. Blockages in the portal vein and inferior vena cava were relieved, followed by blockage of the hepatic artery for 2 h to induce warm ischemia/reperfusion injury. After this period of occlusion, the clamp was removed. Thus, the bile duct ischemia/reperfusion injury model was established.

### Serum Analysis

Blood was collected from the heart via the diaphragm for the measurement of ALT, ALP, DBIL, TNF-α and sFas levels. ALT, ALP and DBIL were determined using commercial kits, with the automatic biochemical analyzer (Olympus-AU5400; Japan) provided by the Laboratory of Southern Hospital of Southern Medical University. Serum TNF-α and sFas were measured using ELISA according to the manufacturer's instructions.

### Caspase-3 Activity Assay

Protein of extrahepatic bile duct were prepared following manufacturer's instruction by using Bradford Protein Assay kit (Beyotime Institute of Biotechnology, Nantong, Jiansu, China) in which tissue were mixed with Ac-DEVD-pNA substrate for 2 h at 37°C prior to colorimetric measurement of *p*-nitroanilide product at 405 nm.

### Pathological Examination

The tissue of extrahepatic bile duct preserved in 5% paraformaldehyde was taken, cropped and made into wax pattern, thereafter sliced up, dyed with hematoxylin-eosin. Edema of bile duct cells, infiltration of inflammatory cells were observed with high power field microscope. With the modified method, the sections of bile duct were assessed for pyknosis, cell necrosis and cell shedding. Histological indicators were analyzed by the semiquantitative assessment of changes from score 0 to 3, in which zero represented normal tissue, and the score increased gradually from 1 (minimal changes) to 3 (severe alteration from normal tissue).

### TUNEL Staining

The concentration of apoptotic cells was determined by TUNEL staining of extrahepatic bile duct sections according to the manufacturer's instructions. Extrahepatic bile duct was cut into 4-µm section. Following TUNEL staining, cells with brown nuclei were recognized as positive. Apoptotic cells were counted at magnification ×400 in 5 randomly and blindly chosen fields per section. The apoptotic index (AI) was defined as the percentage of apoptotic cells among the total number of cells.

### Immunohistochemistry

Immunohistochemical analysis of Fas was performed overnight using anti-Fas polyclonal rabbit antibody (Santa Cruz Biotechnology, USA) at a dilution of 1∶100 at 4°C. A Polinl-2 Plus HRP rabbit/mouse kit (Zhongshan Goldenbridge Biotech Co., Ltd, China) was used according to the manufacturer's instructions. Immunoperoxidase staining was performed with the diaminobenzidine substrate kit (Zhongshan Goldenbrideg Biotech Co., Ltd). Negative control slides were prepared without the primary antibody while still performing all the other steps. Brown–yellow cytoplasmic granules were recognized as positive staining. The number of positive cells was counted in 5 random microscopic fields under the light microscope at a magnification of ×400. Relative protein levels were presented as the percentage of positive cells among the total number of cells.

### Statistical Analysis

All data are presented as mean ± standard deviation. Statistical analysis was performed using analysis of variance when comparing three groups, followed by the Fisher's least significant different test. Values were analyzed using the statistical package SPSS for Windows version 13.0 (SPSS Inc., Chicago, IL, USA). *P* values <0.05 were considered statistically significant.

## Results

### Serum Transaminases

**Figure 1:**
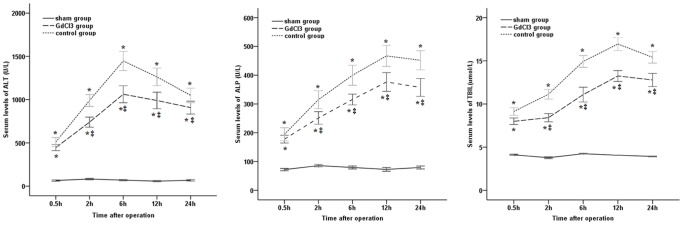
Serum transaminases concentrations in different groups at different time points. Serum levels of ALT, ALP and TBIL were dramatic increased at the early stage of warm ischemia/reperfusion injury. Serum transaminases concentrations were decreased by preadminstration of GdCl_3_. ^*^Significant increase compared with the sham group (*P*<0.05); ^‡^statistically significant difference between the GdCl_3_ and control groups (*P*<0.05). Error bars represent standard deviations.

Serum ALT, ALP and DBIL concentrations at different time points are shown in Fig. 1. Those for ALT, ALP and DBIL levels were significantly higher in the control group than in the sham group at all indicated time points (*P*<0.01); Bile duct function was evaluated with ATL, ALP and TBIL, respectively. In most case, the damage was most evident in control group. Pretreatment with GdCl_3_ significantly alleviated the damage at 2, 6, 12 and 24 h after operation (*P*<0.05).

### Serum TNF-α and sFas levels

**Figure 2:**
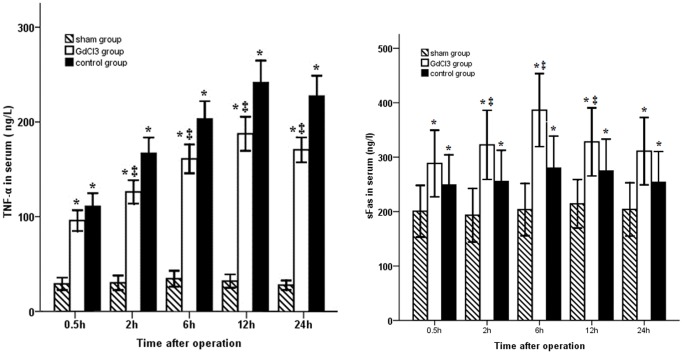
Time course of changes in the TNF-α and sFas levels. TNF-α was significantly increased and reached a peak at 12 h in both ischemia/reperfusion groups. TNF-α was significantly lower in the GdCl_3_ group than in the control group except at 0.5 h, while serum sFas level was increased in animals with ischemia/reperfusion injury. Preadminstration of GdCl_3_ led to a significant increase in sFas level between 2 and 12 h after operation.

As shown in Fig. 2, the serum level of TNF-α was markedly increased during warm ischemia/reperfusion, reaching a plateau 12 h after operation. Pretreatment with GdCl_3_ significantly decreased this level at 2, 6, 12 and 24 h after operation (*P*<0.05). In addition, the sFas level was increased in the animals induced with bile duct warm ischemia/reperfusion injury. Preadminstration of GdCl_3_ led to a significant increase in sFas levels between 2 and 12 h after operation (*P*<0.05).

### Caspase-3 Activity

At 2, 6, 12 and 24 h after operation, the Caspase-3 activity in GdCl_3_ group was lower than in the control group (*P*<0.05), but there was no significant difference between the GdCl_3_ and control groups at 0.5 hour. The Caspase-3 activity of both groups gradually increased and reached a peak between 6 and 12 hours (Table 1).

**Table 1:** The Caspase-3 activity in the three experimental groups at different time points.

Group	Time point (h)				
	0.5	2	6	12	24
Sham	5.80±1.37	6.45±0.53	5.84±0.93	5.67±1.01	6.34±1.12
GdCl_3_	7.12±1.78	8.69±2.33‡	10.16±2.30* ‡	11.69±1.99* ‡	11.31±3.14* ‡
Control	9.54±1.98*	11.98±2.21*	14.12±1.21*	15.92±2.66*	15.03±2.59*
*F*	4.50	7.38	22.73	21.39	10.34
*P*	0.04	0.01	<0.01	<0.01	<0.01

*P*<0.05 was considered significant.

* Significant increase compared with the sham group;

‡ significant reduction compared with the control group.

### Apoptosis of Bile Duct Cell

The bile duct sections were stained by the TUNEL method to evaluate cell apoptosis. The number of TUNEL-stained cells increased in the ischemia/reperfusion groups compared with sham group (*P*<0.05). Administration of GdCl_3_ prevented the increase in bile duct apoptosis at 2, 6, 12 and 24 h after surgery (*P*<0.05) (Table 2).

**Table 2:** Apoptosis of bile duct cells in the three experimental groups at different time points.

Group	Time point (h)				
	0.5	2	6	12	24
Sham	2.06±0.78	2.37±0.27	2.29±0.29	2.32±0.21	2.30±0.20
GdCl_3_	14.68±1.06*	16.99±1.39* ‡	18.28±1.68* ‡	17.31±1.23* ‡	16.54±1.11* ‡
Control	16.48±1.70*	21.83±1.93*	25.46±2.16*	24.98±2.06*	24.56±1.78*
*F*	124.23	159.98	168.59	208.99	261.79
*P*	<0.01	<0.01	<0.01	<0.01	<0.01

Quantitative analysis of TUNEL-stained cell was conducted by apoptosis index (AI).

* Significant increase compared with the sham group;

‡ significant reduction compared with the control group.

### Expression of Fas Protein

**Figure 3:**
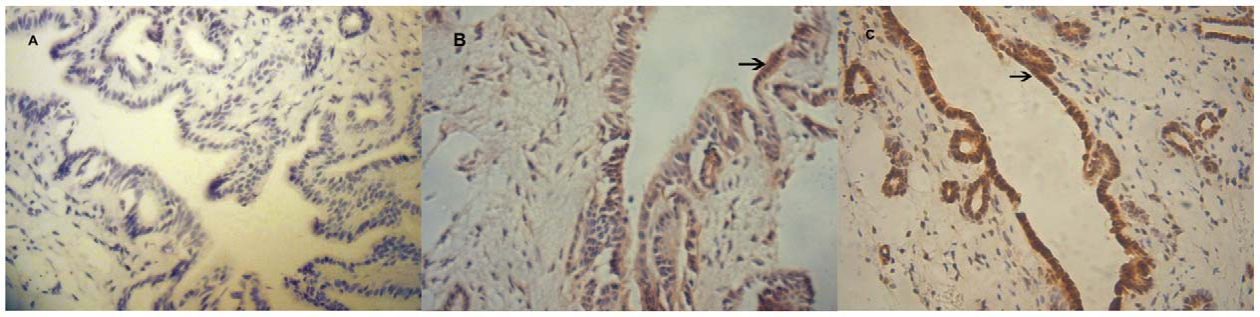
Immunohistochemical detection of Fas in the sham and ischemia/reperfusion groups. Paraffin-embedded sections from the sham (A), GdCl_3_ (B), and control groups (C) at 6 h following ischemia/reperfusion were reacted with anti-Fas serum. Arrows indicate Fas-positive cells (magnification ×400).

**Figure 4:**
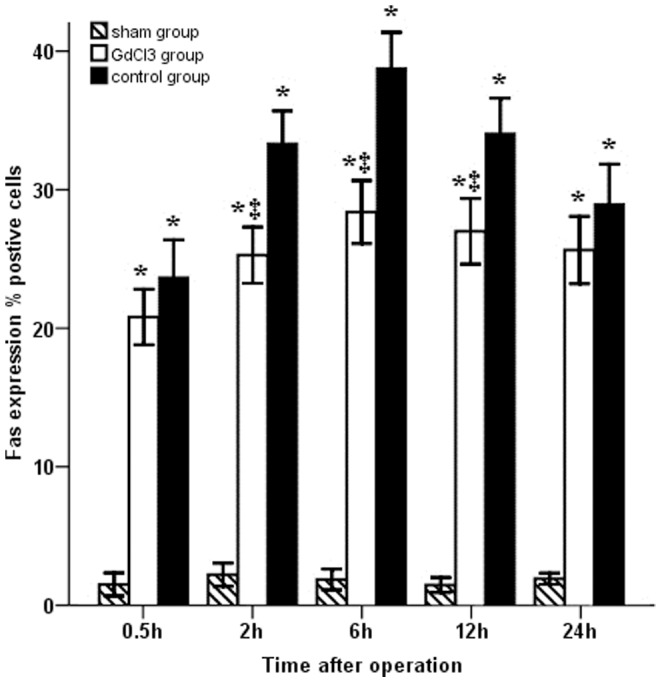
Distribution of Fas-positive cells in serial bile duct samples. The percentage of positive cells in GdCl_3_ and control groups increased slowly for the first 6 hours and reached a peak at 6 hour, but the rate of increase in GdCl_3_ group was slower than in the control group.

To elucidate the mechanisms of apoptosis, the concentration of apoptotic Fas protein was measured by immunohistochemistry in all three groups (Fig. 3). Warm ischemia/reperfusion injury induced an increase in the number of Fas-positive cells compared with the sham group at all indicated time points (*P*<0.05). Following pretreatment with GdCl_3_, the expression of Fas was significantly lower than that in the control group at 2, 6 and 12 h after surgery (*P*<0.05), but was higher than that in the sham group (Fig. 4).

### Pathological Examination

**Figure 5:**
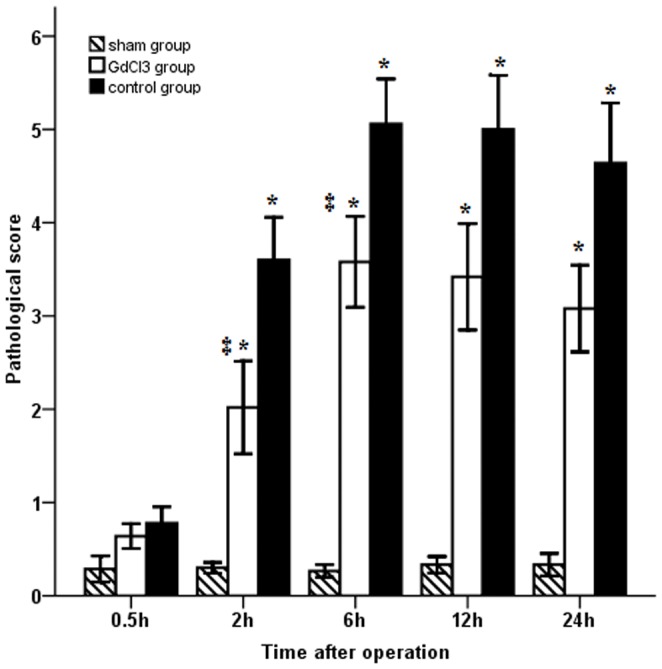
Comparison of pathomorphology in different groups. In control and GdCl_3_ groups, the main injury of bile duct was at 6 hour after surgery. Pretreatment with GdCl_3_ may attenuate the bile duct injury during the ischemia/reperfusion.

Statistical significance was found among groups. The bile duct showed a normal appearance in sham group, and more marked histological changes occurred in control group. The injury at 6 hour after operation was more serious than other time points (Fig. 5).

## Discussion

Recent studies have demonstrated that GdCl_3_ can prevent alcoholic liver injury, minimize ischemia/reperfusion injury, and prevent primary graft dysfuction after experimental liver transplantation [10], [15], [16]. However, the mechanism involved is still incompletely understood; it may be partly related to cell apoptosis. In this study, we demonstrated by the TUNEL method that the number of apoptotic bile duct cells increased markedly in the early stage of warm ischemia/reperfusion injury, and these number decreased markedly after GdCl_3_ treatment. These findings suggest that GdCl_3_ plays an important role in suppressing bile duct cell apoptosis during the early stage of warm ischemia/reperfusion injury.

Serum enzymes are key indicators with regard to bile duct cells injury. In the present study, ALT, ALP and TBIL were markedly elevated during the first 12 h, and then slowly decreased, and the levels of ALT, ALP and TBIL were significantly increased in the control group. Likewise, in biliary pathomorphologic changes, the biliary tract injury in GdCl_3_ group was less serious than that in the control group. The analysis of serum enzymes and pathomorphologic changes showed that the bile duct ischemia/reperfusion injury took place in the early stage, but recovery occurred in the middle and late stage. Meanwhile, the administration of GdCl_3_ resulted in a significant decrease in serum enzyme activity. As we known, KC participates in many pathophysiologic responses after reperfusion, including the release of activation factors and free radicals. The activation factors and free radicals may interaction to decrease the prostaglandin production at that stage. These changes then activated phospholipase A_2_, resulting in endothelial cells degeneration and blood coagulation in the biliary tract microcirculation [19]. In addition, since the blood supply to the extrahepatic bile duct is derived solely from the hepatic artery [20], bile duct injury was aggravated.

Likewise, in biliary pathomorphologic changes, the bile duct injury in GdCl_3_ group was less serious than that in the control group. To further elucidate the precise mechanism of GdCl_3_, we investigated the regulators of apoptosis. Raised TNF-α levels strongly induced apoptosis and necrosis [10], [13], while TNF-α triggered leukocyte chemotaxis and activated many of the proteins involved in apoptosis, such as the proteases caspase-3 and caspase-8 [21]. Activated caspase-3, along with mitochondria cytochrome-C released into the cytoplasm, cleaved cellular substrates to morphological changes in cells and nuclei during apoptosis [22]. Our data demonstrated that TNF-α was overproduced during the early phase of warm ischemia/reperfusion injury while pretreatment with GdCl_3_ significantly down-regulated its production, roughly in accordance with the findings from the TUNEL assay. With regard to liver transplantation, KC have been identified as the critical source of TNF-α [23]. GdCl_3_ competitively binded with the KC calcium receptor to block the activity of nuclear factor-kappa B (NF-κB), consequently inhibiting the transcription of TNF-α [10], [13], [14], [24].

The Fas-Fas ligand is another pathway associated with bile duct cell apoptosis during warm ischemia/reperfusion. An increase in sFas inhibits apoptosis in Fas-expressing cells by binding either the Fas ligand (FasL) or membrane-bound Fas or by interacting with other proteins expressed on these cells [25]. In this study, our data showed that pretreatment with GdCl_3_ clearly increased sFas levels in rats with ischemia/reperfusion injury. A correlation was also found between TUNEL assay and serum sFas levels. The results demonstrated that GdCl_3_ may activate a negative feedback mechanism during warm ischemia/reperfusion injury by increasing sFas, aimed at prevention of additional cell loss.

To further explore the roles of GdCl_3_ in bile duct apoptosis, we measured Fas protein by immunohistochemistry. Fas-mediated apoptosis is regarded as an important effector process in progressive bile duct loss [26]. Binding of Fas to its ligand results in receptor cross-linking and apoptosis of Fas-positive cells via cellular pathways, including receptor oligomerization and recruitment of the Fas-associated protein with the death domain, which mediates the activation of the proteolytic activity of caspase-8 and other downstream caspases, such as caspase-3 [7], [27]. In the present study, Fas immunoreactivity was seen in both ischemia/reperfusion groups, and its expression was more markedly enhanced in the control group than in the GdCl_3_ group. The caspase-3 results conformed roughly with the apoptosis from the TUNEL method. After the operation for 2, 6, 12 and 24 hours, the Caspase-3 activity in GdCl_3_ group was lower than in the control group. GdCl_3_ suppressed the expression of Fas in bile duct cells during ischemia/reperfusion; this may be related to the suppression of cytokine-induced KC activation. As mentioned above, the apoptotic process involves various genes, proteins, and activation factors. In the study, pretreatment with GdCl_3_ may largely decrease the caspase-3 activity, and some researchers have implied that caspase-3 cleavage is strongly augmented soon after reperfusion of liver grafts [10]. So we conjecture GdCl_3_ could depress the expression of Fas protein and caspase-3 activity to suppress bile duct apoptosis.

In summary, the present study demonstrated that GdCl_3_ regulates bile duct apoptosis after warm ischemia/reperfusion injury. These effects may be related to the suppression of the Fas-FasL pathway and the inhibition of the caspase-3. These findings provide strong evidence that GdCl_3_ protects the rat bile duct against early warm ischemia/reperfusion injury during liver transplantation.
